# Supplementary material for: MetaQTL: a package of new computational methods for the meta-analysis of QTL mapping experiments
Source: BMC Bioinformatics. 2007 Feb 8;8:49. doi: 10.1186/1471-2105-8-49 (PMC1808479; doi:10.1186/1471-2105-8-49)
Supplement: Additional File 7 — MetaQTL Package : jar file and tutorial. This Zip archive contains both the MetaQTL JAR file and the files of the tutorial. [file 1471-2105-8-49-S7.zip › org.inra.metaqtl/doc/index-files/index-17.html]

R-Index


|  |  |  |
| --- | --- | --- |
| **Overview** \| Package \| Class \| Use \| **Tree** \| **Deprecated** \| **Index** \| **Help** | |  |
| **PREV LETTER**   **NEXT LETTER** | **FRAMES**    **NO FRAMES**     **All Classes** |  |


A B C D E F G H I K L M N O P Q R S T U V W X Y Z 

---


## **R**

**r2R()** - Method in class org.metaqtl.Chromosome: **r2R(int, int)** - Method in class org.metaqtl.Chromosome: Re-scale the chromosome assuming that the distances and recombination fractions are based on the frequency of recombinant genotypes R instead of the real recombination fraction r. **R2r()** - Method in class org.metaqtl.Chromosome: **R2r(int, int)** - Method in class org.metaqtl.Chromosome: Re-scale the chromosome assuming that the distances and recombination fractions are based on the frequency of recombinant genotypes R instead of the real recombination fraction r. **R2r(double, int, int)** - Static method in class org.metaqtl.util.OffspringManager: This routine returns the recombination rate r for a given value of the propertion of recombibant genotypes R according to the cross design. **r2R(double, int, int)** - Static method in class org.metaqtl.util.OffspringManager: This routine returns the recombination rate r for a given value of the propertion of recombibant genotypes R according to the cross design. **rate** - Variable in class org.metaqtl.EMResult: The quadratric rate of convergence of the algorithm. **read(Reader)** - Static method in class org.metaqtl.factory.DubiousLocusFactory: This routine reads the list of dubious markers from the given file location and returns if succeed an array of `IBioLocus` objects. **read(BufferedReader)** - Static method in class org.metaqtl.factory.EMResultFactory: **read(Reader)** - Static method in class org.metaqtl.factory.MapDBFactory: This method reads a file formated as follows : map property1 property2 property3 ... **read(Reader)** - Static method in class org.metaqtl.factory.MetaDicoFactory: **read(InputStream)** - Static method in class org.metaqtl.factory.MetaQtlAnalysisFactory: **read(Reader)** - Static method in class org.metaqtl.factory.MetaQtlAnalysisFactory: **read(Reader)** - Static method in class org.metaqtl.factory.MetaQtlModelFactory: This method reads a file formated as follows : Chromosome \t Trait \t Model **read(Reader)** - Static method in class org.metaqtl.factory.TreeFactory: **read(String)** - Static method in class org.metaqtl.factory.TreeFactory: **read\_newick(Reader)** - Static method in class org.metaqtl.factory.TreeFactory: **read\_newick(String)** - Static method in class org.metaqtl.factory.TreeFactory: **rebuildPartition(String[])** - Method in class org.metaqtl.QtlPartition: **recombination(double, int, int)** - Static method in class org.metaqtl.util.MappingFunction: Returns the recombination rate from the distance. **removeAllele(String)** - Method in class org.thalia.bio.entity.AlleleContainer: **removeAllele(String)** - Method in interface org.thalia.bio.IBioLocus: **removeGroup(String)** - Method in class org.thalia.bio.entity.GroupContainer: **removeGroup(String)** - Method in interface org.thalia.bio.IBioGenome: **removeIndividual(String)** - Method in class org.thalia.bio.entity.IndividualContainer: **removeIndividual(String)** - Method in interface org.thalia.bio.IBioPopulation: **removeLocus(IBioGenome[], String)** - Static method in class org.metaqtl.main.MetaMain: **removeLocus(String)** - Method in class org.thalia.bio.entity.LocusContainer: **removeLocus(String)** - Method in interface org.thalia.bio.IBioLGroup: **ResourceBioEntityQuery** - Class in org.thalia.bio.util: This class makes it possible to make request on resource content using an array of requested entities. **ResourceBioEntityQuery()** - Constructor for class org.thalia.bio.util.ResourceBioEntityQuery: **resultByChrom** - Variable in class org.metaqtl.MetaQtlAnalysis: The matrix of results where the rows are the chromosomes. **RF** - Static variable in interface org.thalia.bio.IBioCrossTypes: **RIGHT\_LABEL** - Static variable in class org.metaqtl.graph.ChromLayer: **RIGHT\_LABEL** - Static variable in class org.metaqtl.graph.ChromShape: **RILSe** - Static variable in interface org.thalia.bio.IBioCrossTypes: **RILSi** - Static variable in interface org.thalia.bio.IBioCrossTypes: **root** - Variable in class org.metaqtl.Tree: The root of the tree. **root** - Variable in class org.thalia.bio.entity.bean.OntologyBean: The root of the ontology. **rsd** - Variable in class org.metaqtl.MetaChrom: The standardized residual between original marker intervals and those of the meta chromosome. **rsd** - Variable in class org.metaqtl.MultiFitResult: The residual standard deviations. **rsquare** - Variable in class org.metaqtl.Qtl.QTLStats: The r-square, i.e the proportion of variance explained by the QTL. **rtsafe(NumericalUtilities.NRFunction, double, double, double)** - Static method in class org.metaqtl.numrec.NumericalUtilities: **run(Chromosome, Chromosome)** - Static method in class org.metaqtl.algo.CMSAlgorithm: This methods find the common marker sequences between the two given chromosomes. **run(double[], double[])** - Static method in class org.metaqtl.algo.HClustAlgorithm: This method performs a agglomerative clustering of the given data points where `x` is the vector of observed values and `sd` the vector of their standard deviations. **run()** - Method in class org.metaqtl.algo.MetaMapAlgorithm: This method builds a consensus genetic map from the given array of `maps`. **run()** - Method in class org.metaqtl.algo.QtlClustAlgorithm: This method performs a QTL meta-analysis on each chromosome of the given map. **run()** - Method in class org.metaqtl.algo.QtlProjAlgorithm: This routine performs the projection of the QTL from the maps onto the skeleton and returns the chromosomes on which the projection has been done. **run()** - Method in class org.metaqtl.algo.QtlTreeAlgorithm: This method performs an agglomerative hierarchical QTL clustering on each chromosome and returns the result as a `MetaQtlAnalysis` object. **run()** - Method in interface org.metaqtl.IMetaAlgorithm: Run the algorithm.

---


|  |  |  |
| --- | --- | --- |
| **Overview** \| Package \| Class \| Use \| **Tree** \| **Deprecated** \| **Index** \| **Help** | |  |
| **PREV LETTER**   **NEXT LETTER** | **FRAMES**    **NO FRAMES**     **All Classes** |  |


A B C D E F G H I K L M N O P Q R S T U V W X Y Z 

---
